# Performance of the recommended ESC/EASD cardiovascular risk stratification model in comparison to SCORE and NT-proBNP as a single biomarker for risk prediction in type 2 diabetes mellitus

**DOI:** 10.1186/s12933-021-01221-w

**Published:** 2021-02-02

**Authors:** Suriya Prausmüller, Michael Resl, Henrike Arfsten, Georg Spinka, Raphael Wurm, Stephanie Neuhold, Philipp E. Bartko, Georg Goliasch, Guido Strunk, Noemi Pavo, Martin Clodi, Martin Hülsmann

**Affiliations:** 1 Department of Internal Medicine II, Medical University of Vienna, Waehringer Guertel 18-20, 1090 Vienna, Austria; 2 Department of Internal Medicine, Saint John of God Hospital Linz, Seilerstaette 2, 4021 Linz, Austria; 3 Department of Medicine IV, Clinic Favoriten, Kundratstraße 3, 1100 Vienna, Austria; 4 Complexity Research, Schönbrunner Straße 32, 1050 Vienna, Austria

**Keywords:** Diabetes mellitus, Cardiovascular risk, Prevention

## Abstract

**Background:**

Recently, the European Society of Cardiology (ESC) and European Association for the Society of Diabetes (EASD) introduced a new cardiovascular disease (CVD) risk stratification model to aid further treatment decisions in individuals with diabetes. Our study aimed to investigate the prognostic performance of the ESC/EASD risk model in comparison to the Systematic COronary Risk Evaluation (SCORE) risk model and N-terminal pro-B-type natriuretic peptide (NT-proBNP) in an unselected cohort of type 2 diabetes mellitus (T2DM).

**Methods and results:**

A total of 1690 T2DM patients with a 10-year follow up for fatal CVD and all-cause death and a 5-year follow up for CVD and all-cause hospitalizations were analyzed. According to ESC/EASD risk criteria 25 (1.5%) patients were classified as moderate, 252 (14.9%) high, 1125 (66.6%) very high risk and 288 (17.0%) were not classifiable. Both NT-proBNP and SCORE risk model were associated with 10-year CVD and all-cause death and 5-year CVD and all-cause hospitalizations while the ESC/EASD model was only associated with 10-year all-cause death and 5-year all-cause hospitalizations. NT-proBNP and SCORE showed significantly higher C-indices than the ESC/EASD risk model for CVD death [0.80 vs. 0.53, p < 0.001; 0.64 vs. 0.53, p = 0.001] and all-cause death [0.73, 0.66 vs. 0.52, p < 0.001 for both]. The performance of SCORE improved in a subgroup without CVD aged 40–64 years compared to the unselected cohort, while NT-proBNP performance was robust across all groups.

**Conclusion:**

The new introduced ESC/EASD risk stratification model performed limited compared to SCORE and single NT-proBNP assessment for predicting 10-year CVD and all-cause fatal events in individuals with T2DM.

## Background

Diabetes is associated with a substantially increased risk to develop cardiovascular disease (CVD) [1]; however, as individuals with diabetes represent a highly heterogeneous population, incremental CVD risk is not equally distributed among diabetic patients [2]. Therefore, the development of individualized CVD risk assessment tools is essential to warrant a personalized therapy approach.

Recently, the European Society of Cardiology (ESC) in collaboration with the European Association for the Study of Diabetes (EASD) published new guidelines for the prevention and management of CVD in patients with prediabetes and diabetes [3]. For the first time, the use of a CVD risk stratification model is recommended to aid treatment decisions in individuals with diabetes. The ESC/EASD risk model stratifies diabetic patients into three different risk categories based on the 10-year risk estimate for fatal CVD adapted from the 2016 European Guidelines on CVD prevention in clinical practice [4]. To the best of our knowledge, the predictive performance of the newly introduced risk stratification model has not been verified in individuals with diabetes.

The Systematic COronary Risk Evaluation (SCORE) equation is commonly used for estimating the 10-year risk of fatal CVD in the general population [5]. In the original publication of the SCORE project, it has been suggested that SCORE based risk assessment could be used for a rough assessment of CVD risk in diabetic patients. Yet, the predictive performance of SCORE has not been tested in patients with long-standing diabetes specifically [6]. As a result, its application for risk estimation in individuals with diabetes cannot be recommended [4].

The prognostic value of N-terminal pro-B-type natriuretic peptide (NT-proBNP) for CVD outcomes in patients with diabetes has been demonstrated and was confirmed in numerous studies [7–24]. Nonetheless, the new guidelines do not recommend routine assessment of circulating biomarkers for CVD risk estimation in diabetic patients [3].

This study aimed to perform a head-to-head comparison of the predictive performance of the ESC/EASD model against SCORE and NT-proBNP for risk assessment of 10-year CVD death and all-cause death (i) in an unselected type 2 diabetes mellitus (T2DM) cohort, (ii) in selected patients with T2DM with characteristics similar to the SCORE derivation cohort and (iii) to investigate the outcome-specific performance of the different risk estimates. Additionally, the prognostic utility of the risk assessments for 5-year CVD and all-cause hospitalization was evaluated.

## Methods

### Study population

From December 2005 through January 2010 a total of 2186 patients with T2DM from 4 diabetes outpatient clinics were included in a prospective registry. Medical history, including comorbidities, diabetes duration, medical therapy and assessment of risk factors, was recorded at enrolment. Patients were followed up as clinically appropriate. All patients gave written informed consent. The study was approved by the local Ethics Committee of the Medical University of Vienna and complies with the principles of the Declaration of Helsinki.

### Laboratory analysis

Blood samples were collected under fasting conditions and immediately sent to the local laboratory. Estimated glomerular filtration rate (eGFR) was assessed by the Modified Diet for Renal Disease Study equation. NT-proBNP determination was performed directly using the cobas h 232 point-of-care analyzer by Roche (Roche Diagnostics, Basel, Switzerland) with a lower detection limit of 59 pg/ml. Within-series coefficients of variation ranged from 4.8 to 14.8% as previously determined by Bertsch et al. [25] Urine albumin/creatinine ratio was assessed quantitively in fresh spot urine samples according to the local laboratory standards.

### Calculating risk estimates

- ESC/EASD risk stratification model
  Patients were categorized as moderate, high and very high risk based on the predicted 10-year risk estimates for CVD death < 5%, 5–10% and > 10%, respectively, according to the ESC/EASD cardiovascular risk categories as indicated in Additional file 1: Table S1. [3] Except for renal impairment no precise definition on the rating of the respective risk factors used within the ESC/EASD stratification model was given. Thus, we defined age, obesity and proteinuria as being at risk > 50 years, ≥ 30 kg/m^2^ and with a urinary albumin/creatinine ratio > 30 mg/mmol, respectively. High blood pressure and dyslipidemia were defined according to the criteria of the respective current European guidelines [26, 27], as documented in medical charts or on specific therapy. Smoking status was assessed based on hospital charts and by self-report. CVD diagnosis was defined with a corresponding main diagnosis according to at least one of the following International Statistical Classification of Diseases and Related Health Problems 10th Revision (ICD-10) codes: I21–I25 (ischemic heart diseases), I63–I66 (cerebral artery disease), I70–I74 (peripheral artery disease), I44.7 (left bundle-branch block), I50 (heart failure) I48 (atrial fibrillation and flutter), I11 (hypertensive heart disease) and I34–I36 (valve disorders). As retinopathy and left ventricular hypertrophy were not systematically assessed, these variables were not included in the analysis. Additional file 1: Table S2 provides an overview of the specific cut-offs and definitions used for risk stratification of the ESC/EASD model.
- SCORE risk model
  SCORE risk estimation is recommended for individuals without CVD and aged 40–64 years in accordance to the selection criteria of SCORE [4, 5]. The 10-year fatal CVD risk was calculated using the low SCORE risk chart based on the risk variables age, sex, smoking status, total cholesterol and systolic blood pressure [5]. As indicated in the reference publication, we multiplied the SCORE risk estimates by 2 for men and 4 for women to account for the increased CV (cardiovascular) risk in individuals with diabetes [5]. Risk estimation for individuals aged < 40 and > 65 years was performed referring to the risk estimates provided for individuals aged 40 and 65 years, respectively.

### Endpoints

The primary outcome measure was CVD death at 10 years. Additional secondary outcome measures were all-cause death at 10 years and unplanned CVD as well as all-cause hospitalization at 5 years. Time at risk was calculated as time between enrolment and event or end of follow-up period whichever came first. Data on death diagnosis was obtained from the Austrian Death Registry which includes information on cause of death based on ICD-10 codes. CVD events resulting in death or hospitalization were defined as atherosclerotic CVD, valvular heart disease, heart failure, malignant arrhythmia, peripheral artery disease and cerebrovascular disease. In case of unclear ICD-classifications, the hospital charts were further examined to give a definitive diagnosis of cause of death. The adjudication of CVD deaths was carried out by an experienced clinician who was blinded for the various risk assessments.

### Statistical analysis

Continuous data are presented as median and interquartile range (IQR) and discrete data as frequency and percentages. Continuous variables were compared by the Kruskal–Wallis and Mann–Whitney-U-test, counts by the Fisher’s exact test.

For the comparison between the risk estimators, i.e. NT-proBNP, ESC/EASD risk model and SCORE, both NT-proBNP and SCORE were entered as continuous as well as categorical variables (NT-proBNP: tertiles and two groups with cut-off: 125 pg/ml; SCORE: 3 risk groups with cut-off: < 5%, 5–10%, > 10%). All comparisons were made for the total cohort and two subgroups similar to the characteristics of the original derivation cohort of SCORE. For the first subgroup individuals with established CVD were excluded and the second subgroup consisted of patients without established CVD and an age ≥ 40 and < 65 years. For outcome analysis we used a cause-specific hazard model. Cox regression was performed to evaluate the association of the risk assessments with 10-year fatal CVD events and secondary outcome measures. Hazard ratios (HRs) of continuous NT-proBNP refer to ln-transformed NT-proBNP per 1-IQR increase. In addition to the univariate analysis, adjustments for potential confounders were conducted to demonstrate the robustness of NT-proBNP. Albumin/creatinine ratio, eGFR and age and body mass index (BMI) were added as continuous variables to the Cox multivariate regression model, while smoking status, hypertension, dyslipidemia, baseline CVD and sex were added as dichotomous variables (yes/no). Proportional hazard assumption was assessed and satisfied for all variables based on time interaction tests. Cumulative incidence plots of the events of interest are shown for the various risk assessments.

Additionally, we performed a competing risk analysis for the Cox regression model for CVD and non-CVD death as competing risks based on the data duplication method introduced by Lunn and McNeil [28]. Proportional hazard assumption for all variables in the augmented data set was assessed and satisfied based on time interaction tests. Therefore, competing risk analysis was performed by using the event variable as a covariate.

Predictive performance was expressed as discrimination (receiver operating characteristic [ROC] curve, Harrell’s C-index) and calibration using Hosmer–Lemeshow goodness-of-fit test. Observed 10-year risk for fatal CVD is presented using the Kaplan–Meier estimates. Differences in outcomes were assessed by non-overlapping confidence intervals (confidence interval [CI] 95%) between C-indices corresponding to p-values of ≤ 0.05.

An improvement in individual risk prediction for the risk assessments was examined by the overall continuous net reclassification improvement (NRI) and presented as NRI (standard error [SE], p-value) as described by Pencina et al. [29] For calculation of the NRI, the ESC/EASD risk stratification was treated as a categorical variable (moderate, high, very high), the SCORE risk model and NT-proBNP as continuous variables. Furthermore, risk classification tables for all reported outcomes (i.e. all-cause mortality, CVD mortality, all-cause hospitalization, CVD hospitalization) were presented comparing SCORE categories and the ESC/EASD risk model with tertiles of NT-proBNP. A two-tailed p-value lower than 0.05 was considered statistically significant. Statistical analysis was performed using SPSS software (IBM SPSS, Chicago, Illinois, USA) version 24, RStudio (R Foundation for Statistical Computing, Vienna, Austria) version 1.3.1073 and STATA software (StataCorp, College Station, Texas, USA) version 13.

## Results

### Study population

A total of 2186 T2DM patients were enrolled in the study, 496 patients were excluded from the analysis as survival status (n = 460) and diabetes duration (n = 36) was not available, thus a total of 1690 T2DM patients were analyzed. Detailed description of the baseline characteristics is displayed in Table 1.

**Table 1:** Baseline characteristics of the overall study cohort

Characteristics	Overall cohort (n = 1690)
Demographics	
Age, years (IQR)	63 [54–69]
Female, n (%)	783 (46)
Diabetes duration, years (IQR)	10 [5–19]
Hypertension, n (%)	1135 (67)
Dyslipidaemia, n (%)	1152 (68)
Smoking, n (%)	339 (20)
BMI, kg/m^2^ (IQR)	28.7 [25.4–32.7]
Cardiovascular disease	
PCI, n (%)	66 (4)
PAD, n (%)	173 (10)
CeVD, n (%)	99 (6)
CABG, n (%)	62 (4)
Medications	
Statins, n (%)	764 (45)
Acetylsalicylic acid, n (%)	634 (38)
Insulin, n (%)	888 (53)
Oral antidiabetics, n (%)	984 (58)
Laboratory parameters	
NT-proBNP, pg/ml (IQR)	122 [59–266]
Albumin/creatinine ratio, mg/mmol (IQR)	0.87 [0.35–2.94]
eGFR, ml/min (IQR)	72.7 [60.3–85.3]
LDL cholesterol, mg/dl (IQR)	102 [82–123]
HbA1c, % (IQR)	7.2 [6.5–8.1]

*BMI* body mass index, *PCI* percutaneous coronary intervention, *PAD* peripheral artery disease, *CeVD* cerebrovascular disease, *CABG* coronary artery bypass graft, *NT-proBNP* N-terminal pro-B-type natriuretic peptide, *eGFR* estimated glomerular filtration rate, *LDL* low-density lipoprotein

Median age of the total study population was 63 years (IQR 54–69), 783 (46%) of the patients were female. CVD was present in 311 patients (18.4%). According to the ESC/EASD risk model criteria, 25 (1.5%) were classified as moderate, 252 (14.9%) as high and 1125 (66.6%) as very high risk. A total of 288 patients (17.0%) were not classifiable based on the stated ESC/EASD criteria, as 280 patients had diabetes duration < 10 years with 1 or 2 (but < 3) established CV risk factors and 8 patients presented with a diabetes duration longer than 10 years without any risk factors. Detailed characteristics for the ESC/EASD risk strata are presented in Additional file 1: Table S3. In the overall cohort, 654 patients (39%) had a calculated SCORE risk estimate below 5%, 525 patients (31%) between 5 and 10% and 511 patients (30%) above 10%. The calculated SCORE risk estimates for 10-year fatal increased with ESC/EASD risk category (0% [IQR 0–0] vs. 6% [IQR 2–10] vs. 8% [IQR 4–12], p < 0.001 between all groups).

**Fig. 1:**
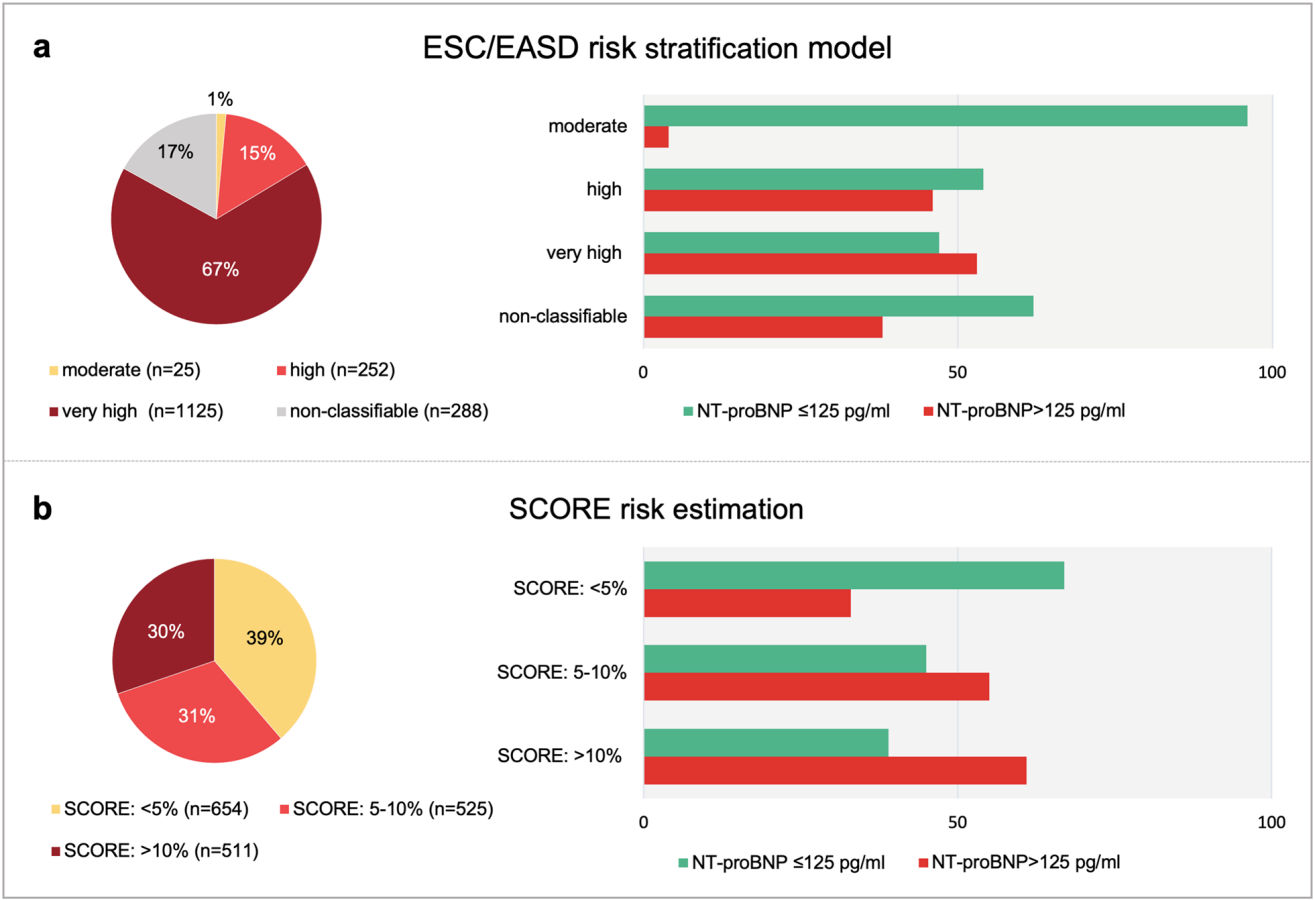
Distribution of the risk estimate as well as the proportion of patients with normal and elevated NT-proBNP (cut-off: 125 pg/ml) for **a** the ESC/EASD risk model and **b** SCORE

Distribution of patients in the ESC/EASD and SCORE risk strata as well as proportion of patients with normal (n = 871) and elevated (n = 819) NT-proBNP levels at a cut-off 125 pg/ml within these groups are illustrated in Fig. 1 (p < 0.001 for both models).

#### Association of the ESC/EASD risk strata, NT-proBNP and SCORE with the primary endpoint CVD death and the secondary outcome all-cause death at 10 years

**Fig. 2:**
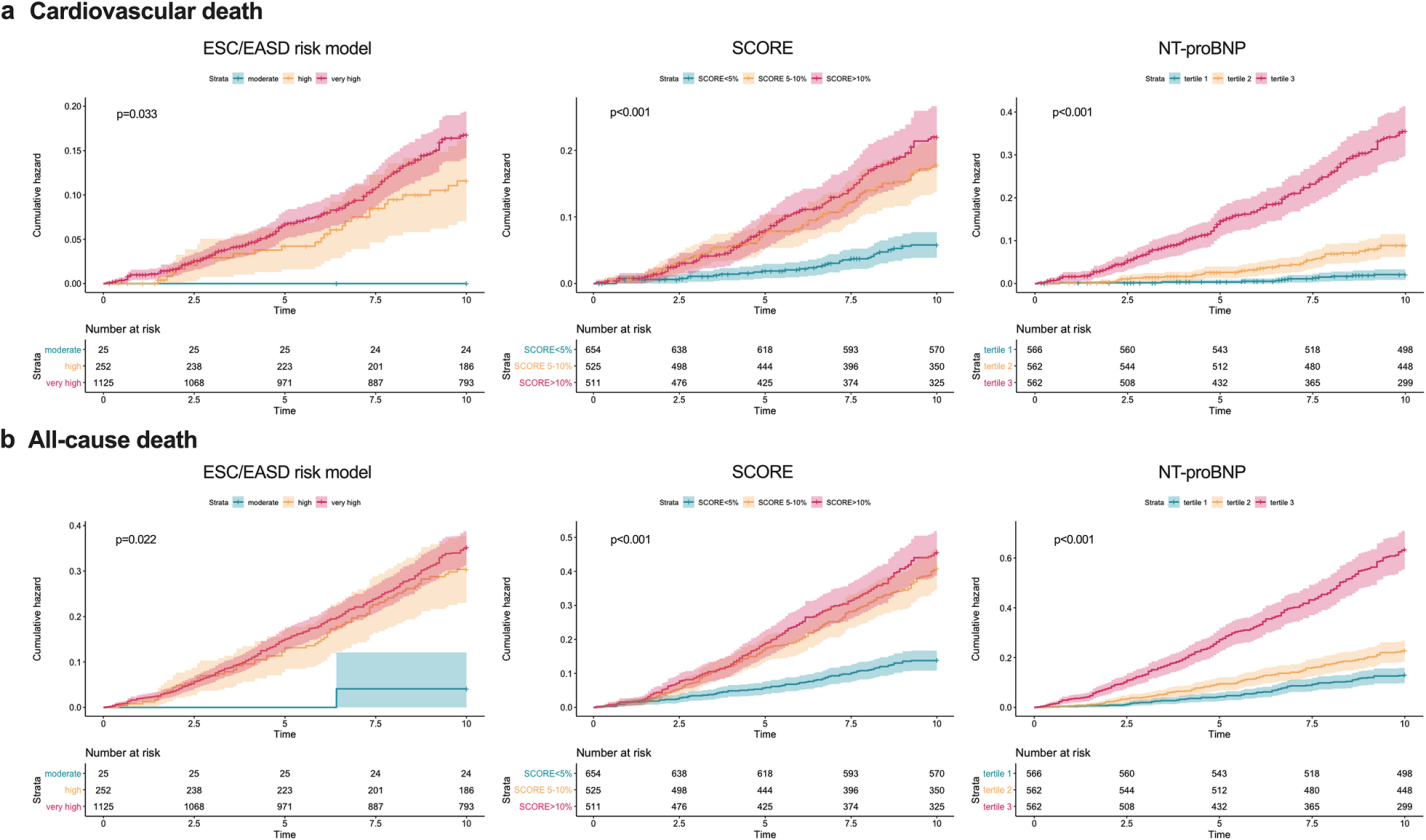
Kaplan–Meier curves showing the cumulative incidence of (**a**) cardiovascular and (**b**) all-cause death for the ESC/EASD risk model (left), SCORE (cut-off: < 5%, 5–10%, > 10%) (middle), and NT-proBNP tertiles (right)

During 10 years of follow-up, 448 patients (26.5%) died, CVD death accounted to 44.9% (n = 201) of all deaths. The cumulative incidences for all risk models stratified into three groups with regards to both endpoints are shown in Fig. 2. SCORE and NT-proBNP were both significantly associated with the primary outcome CVD death and the secondary outcome all-cause death (p < 0.001 for both) while the ESC/EASD risk model was only associated with all-cause death (moderate risk vs. high risk: p = 0.046 and vs. very high risk: p = 0.031). Table 2 shows the results of the univariate Cox regression analysis.

**Table 2:** Association of the ESC/EASD model, NT-proBNP and SCORE with outcome in unselected patients with T2DM (n = 1690)

	10-years cardiovascular death			10-years all-cause death		
	HR **[**95% CI]	P	C-index [95% CI]	HR **[**95%CI]	P	C-index [95% CI]
ESC/EASD risk model	–	–	0.53 [0.50 to 0.56]	–		0.52 [0.50 to 0.54]
Moderate	Reference	–	–	Reference	–	–
High	23.68 [0.08–7013.72]	0.276	–	7.49 [1.04–53.94]	0.046	–
Very high	20.88 [0.25–1747.39]	0.178	–	8.63 [1.21–61.42]	0.031	–
SCORE, %^a^ (< 5%, 5–10%, > 10%)	–	–	0.63 [0.60–0.67]	–	–	0.62 [0.60–0.64]
< 5%	Reference	–	–	Reference	–	–
5–10%	3.10 [2.08–4.63]	< 0.001	–	2.90 [2.24–3.76]	< 0.001	–
< 10%	3.79 [2.56–5.60]	< 0.001	–	3.25 [2.52–4.21]	< 0.001	–
SCORE, %	1.05 [1.04–1.07]	< 0.001	0.64 [0.60–0.67]	1.06 [1.04–1.07]	< 0.001	0.66 [0.63–0.68]
NT-proBNP, tertiles	–	–	0.75 [0.73–0.78]	–	–	0.70 [0.67–0.72]
Tertile 1	Reference	–	–	Reference	–	–
Tertile 2	4.24 [2.19–8.21]	< 0.001	–	1.76 [1.31–2.38]	< 0.001	–
Tertile 3	17.21 [9.3–31.78]	< 0.001	–	4.90 [3.76–6.39]	< 0.001	–
NT-proBNP, pg/ml^b^	5.72 [4.68–7.00]	< 0.001	0.80 [0.77–0.83]	3.32 [2.90–3.79]	< 0.001	0.73 [0.70–0.76]
NT-proBNP, > 125 pg/ml	7.15 [4.85–10.53]	< 0.001	0.71 [0.68–0.74]	3.14 [2.56–3.86]	< 0.001	0.66 [0.63–0.68]

^a^Refers to SCORE treated as categorical variable (cut-off: < 5%, 5–10%, > 10%)

^b^Refers to ln-transformed NT-proBNP per 1-IQR increase

Patients with NT-proBNP > 125 pg/ml had a 7.2-fold and 3.1-fold risk for CVD and all-cause death at 10 years, respectively, compared to individuals with NT-proBNP within the range considered as normal (p < 0.001). NT-proBNP remained a strong predictor of risk irrespective of traditional confounders as age, eGFR, sex, hypertension, smoking, dyslipidaemia, BMI, baseline CVD and albumin/creatinine ratio for both CVD death (ln[NT-proBNP per IQR increase] adjusted HR: 3.96 [2.93–5.35], p < 0.001) and all-cause death (ln[NT-proBNP per IQR increase] adjusted HR: 2.25 [1.85–2.73], p < 0.001).

Competing risk analysis showed qualitatively the same results as in the cause-specific hazard models presented in Table 2. In contrast to SCORE and the ESC/EASD risk model, we found a significant stronger predictive power of NT-proBNP for CVD death than for non-CVD death. Additional file 1: Table S4 provides detailed information on the competing risk analysis.

#### Discriminatory performance of the ESC/EASD risk strata, NT-proBNP and SCORE in unselected T2DM patients

**Fig. 3:**
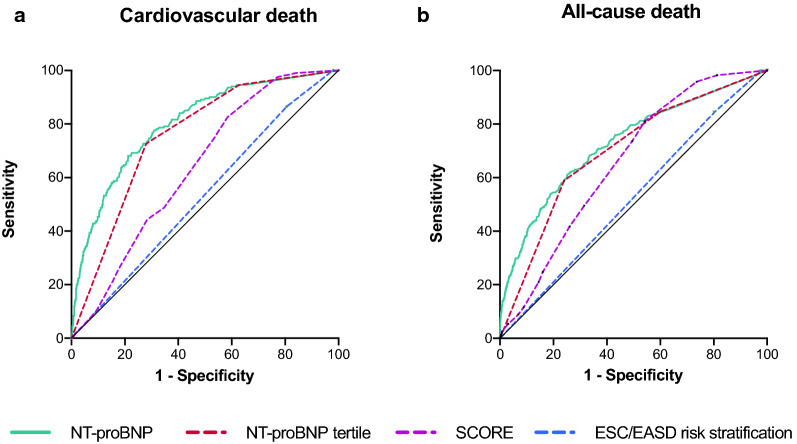
Receiver operating characteristic curves of the ESC/EASD risk model, NT-proBNP and SCORE for the outcomes (**a**) cardiovascular death and (**b**) all-cause death

ROC curves for all risk models and the endpoints 10-year CVD and all-cause death are shown in Fig. 3. Additional file 1: Table S5 provides information on sensitivity, specifity, negative predictive value and positive predictive value of the respective risk assessments. In terms of discriminatory accuracy NT-proBNP was superior to the ESC/EASD risk model for both outcomes (C-index: CVD death: 0.80 vs. 0.53, p < 0.001; all-cause death: 0.73 vs. 0.52, p < 0.001). When NT-proBNP was entered as a categorical variable based on tertiles (1st tertile: 59 pg/mL [IQR 59—59], 2nd tertile: 122 pg/mL [IQR 90—156], 3rd tertile: 376 pg/mL [IQR 267—648]) the results remained virtually unchanged (C-index: CVD death: 0.75 vs. 0.53, p < 0.001; all-cause death: 0.70 vs. 0.52, p < 0.001). Similarly, SCORE showed significantly higher C-indices as compared to the ESC/EASD risk model (C-index: CVD death: 0.64 vs. 0.53, p = 0.001; all-cause death: 0.66 vs. 0.52, p < 0.001). Net individual risk prediction significantly improved when assessing NT-proBNP with the ESC/EASD model with a continuous overall NRI of 0.61 (SE 0.60, p < 0.001) for all-cause death and of 0.74 (SE 0.08, p < 0.001) for CVD mortality. When assessing SCORE with the ESC/EASD model, net individual risk prediction did not improve with a continuous overall NRI of 0.12 (SE 0.08, p = 0.133) for CVD mortality and 0.06 (SE 0.06, p = 0.304) for all-cause mortality.

Furthermore, a detailed reclassification table comparing NT-proBNP tertiles with the ESC/EASD model as well as the SCORE model for all presented outcomes is given in Additional file 1: Table S6.

Additional file 1: Table S7 provides information on the predictive performance of NT-proBNP when added to a baseline model encompassing classical CV risk factors. Also here, NT-proBNP provided prognostic information beyond traditional risk factors.

#### Predictive performance of the ESC/EASD risk strata, NT-proBNP and SCORE in distinct subgroups

The following subgroups were investigated: (i) patients without CVD and (ii) patients without CVD and aged 40–64 years according to SCORE derivation cohort.

Cox regression analysis for the subgroups regarding 10-year fatal outcome are shown in Additional file 1: Table S8. NT-proBNP and SCORE were equally associated with 10-year CVD and all-cause death in subgroups of patients without established CVD and in patients without CVD and aged between 40 and 64 years (p < 0.001 for all). The ESC/EASD risk model was only associated with 10-year all-cause death in patients without CVD when comparing the moderate risk with the high risk category (p = 0.046).

**Fig. 4:**
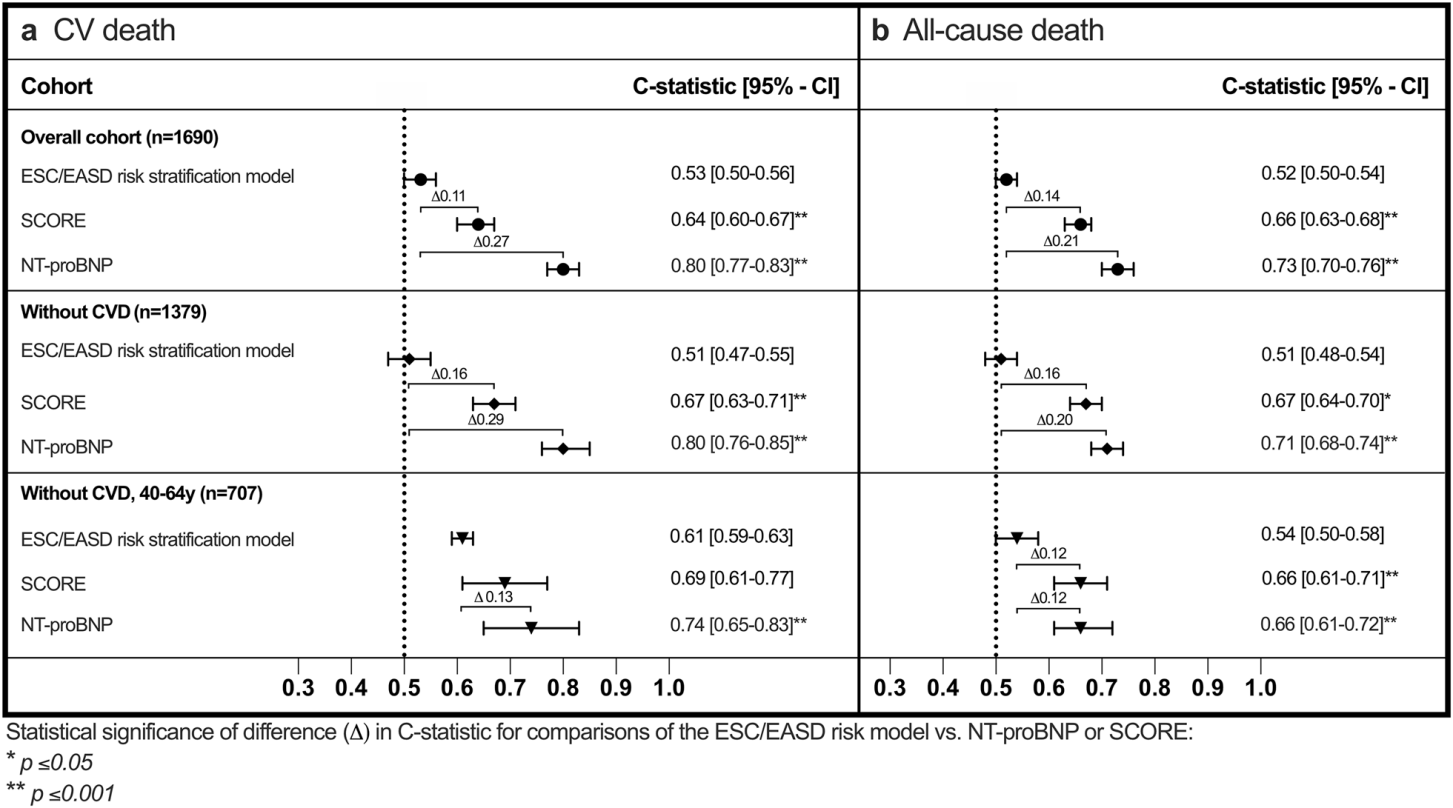
Discriminative performance of the ESC/EASD risk model, NT-proBNP and SCORE in the overall cohort in T2DM individuals without CVD and without CVD aged 40–64 years for the outcome (**a**) cardiovascular (CV) death and (**b**) all-cause death. The figure displays C-indices and 95% confidence intervals (CI)

Figure 4 displays C-statistics of the risk assessments according to the T2DM population studied, the respective ROC graphics are shown in Additional file 1: Figure S1. NT-proBNP was characterized by robustly highest C-indices across both subgroups comparable to the unselected cohort for both endpoints. The ESC/EASD model was characterized by poor C-indices in all groups. The performance of the SCORE risk prediction model improved with progressing exclusivity of patient criteria, performing best in the cohort closest to its derivation population, i.e. in patients without CVD and aged 40–64 years. Both NT-proBNP and SCORE outperformed the ESC/EASD model with regards to CVD death and all-cause death in the subgroup without CVD while only NT-proBNP achieved a significantly higher C-index to predict CVD death in the subgroup including patients without CVD and aged 40–64 years.

#### Observed versus predicted risk estimate for 10-year fatal CVD by SCORE

The SCORE risk algorithm underestimated the actual risk of CVD death in unselected T2DM patients (observed vs. predicted CVD fatal risk: 13% vs. 8% [IQR 4–12]; X^2^ = 70.1, p < 0.001) and in T2DM patients without CVD (observed vs. predicted CVD fatal risk: 10% vs. 8% [IQR 4–12]; X^2^ = 19.8, p < 0.001). When investigating only individuals without CVD and aged 40–64 years, the observed risk was 6% compared with a median predicted risk of 4% [IQR 2–8]. Here, goodness-of-fit for SCORE risk estimate was good with a X^2^ of 4.3 (p = 0.234).

#### Predictive performance of the ESC/EASD risk strata, NT-proBNP and SCORE for the secondary outcomes 5-year CVD and all-cause hospitalization

**Fig. 5:**
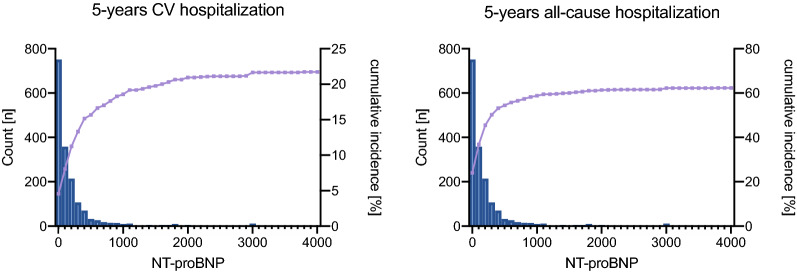
Distribution of NT-proBNP in patients with T2DM and the cumulative incidence of 5-year CVD and all-cause hospitalization

Over a follow-up of 5 years, 1053 (62.3%) patients were hospitalized due to any causes and 367 (21.7%) patients due to unplanned CVD events. Risk for all-cause hospitalization increased by 7% and for CVD hospitalization by 12% per 100 pg/ml increase in NT-proBNP (p < 0.001 for both). Similarly, SCORE was associated with increased risk for CVD and all-cause hospitalizations (p < 0.001 for both) while the ESC/EASD risk stratification model was only associated with all-cause hospitalizations (moderate risk vs. high risk: p = 0.033 and vs. very high risk: p = 0.002). Cox regression analysis is presented in Additional file 1: Table S9. Figure 5 presents cumulative incidence of 5-year hospitalizations for NT-proBNP. In terms of discriminatory accuracy NT-proBNP was superior to the ESC/EASD risk model for both outcomes (C-index: CVD hospitalization: 0.74 vs. 0.54; all-cause hospitalization: 0.62 vs. 0.55; p < 0.001 for all comparisons). In comparison to the ESC/EASD risk model, SCORE showed significantly higher C-indices for CVD-hospitalizations (0.62 vs. 0.54, p = 0.003) and for all-cause hospitalizations (0.59 vs. 0.55, p = 0.040).

The NRI significantly improved when assessing NT-proBNP with the ESC/EASD model with a continuous overall NRI of 0.32 (SE 0.06, p < 0.001) for all-cause hospitalizations and of 0.70 (SE 0.06, p < 0.001) for CVD hospitalizations. Similarly, when assessing SCORE with the ESC/EASD model, net individual risk prediction significantly improved with a continuous overall NRI of 0.15 (SE 0.06, p = 0.008) for all-cause hospitalizations and of 0.14 (SE 0.06, p = 0.024) for CVD hospitalizations.

#### Outcome specifity of NT-proBNP, the ESC/EASD risk strata and SCORE

As indicated by C-statistics, NT-proBNP yielded better discrimination for CVD than for all-cause death (0.80 vs. 0.73, p ≤ 0.05) and for CVD than for all-cause hospitalization (0.74 vs. 0.62, p ≤ 0.05). No difference for the ESC/EASD risk strata (CVD vs. all-cause death: 0.53 vs. 0.52, p > 0.05; CVD vs all-cause hospitalization: 0.54 vs. 0.55, p > 0.05) or the SCORE risk estimation (CVD vs. all-cause death: 0.64 vs. 0.66, p > 0.05; CVD vs all-cause hospitalization: 0.62 vs. 0.59, p > 0.05) could be observed.

## Discussion

This is the first study evaluating the predictive performance of the recently published ESC/EASD risk stratification model in a reasonably large real-world cohort of patients with T2DM and directly comparing this risk model with SCORE and the biomarker NT-proBNP. Our results demonstrate that (i) the ESC/EASD risk stratification model performs limited compared to SCORE and NT-proBNP in terms of risk prediction and discriminatory accuracy (ii) application of SCORE in a selected subgroup of T2DM patients resulted in a similar discriminative ability as achieved in non-diabetics, (iii) in both unselected and selected T2DM patients NT-proBNP remains a robust predictor for outcome, (iv) in contrast to NT-proBNP the ESC/EASD and SCORE risk model showed no outcome specifity for future CVD events in T2DM individuals.

The 2016 ESC guidelines on CVD prevention classify most patients with T2DM at high or very high risk [4]. Although diabetes has long been considered as a “cardiovascular risk equivalent” [30], more recent data indicate that incremental CV risk does not uniformly affect all patients with T2DM [31, 32]. Therefore, tools advocating a more individualized risk assessment are mandatory. The recently updated guidelines for the management and prevention of CVD risk in individuals with diabetes integrated the aforementioned approach and introduced a new risk model accounting also for individuals at moderate risk [3]. The new guidelines recommend for the first time the use of a risk stratification model based on three risk categories (moderate/high/very high) to aid treatment decisions in diabetes while the assessment of biomarkers (e.g. NT-proBNP) is not recommended. However, the predictive performance of the ESC/EASD has neither been derived nor tested in patients with diabetes.

When applying the ESC/EASD risk criteria to our cohort, most patients with 67% were stratified to the very high risk category, 15% met the ESC/EASD criteria for the high risk category whereas the moderate risk category was poorly represented with 1.5%. Moreover, a significant proportion of patients (17%) could not be categorized into either of the ESC/EASD CVD risk categories based on the model’s stratification criteria. Apparently, the newly defined moderate risk category, determined by short diabetes duration and no risk factors at all, is poorly represented in a typical cohort of patients with T2DM. The high prevalence of risk factors such as obesity, hypertension and dyslipidemia even in individuals with short T2DM duration (as part of the metabolic syndrome) may explain the small number of patients stratified into this group [33]. Moreover, our data indicate that the ESC/EASD risk model does not provide an accurate risk estimate to predict 10-year fatal CVD outcome in individuals with diabetes. In direct comparison, both NT-proBNP and SCORE outperformed the ESC/EASD model in terms of risk prediction and discriminatory accuracy.

SCORE based risk estimation has been developed in the general population, but notably the original derivation cohort included also diabetic patients [5]. Since data on diabetes has not been collected uniformly in the SCORE project, the presence of diabetes has not been included as a predictor variable in the risk algorithm. However, it has been suggested that SCORE could be used for a rough risk assessment in patients with diabetes. In the current report, SCORE was significantly associated with 10-year risk of fatal CVD but underestimated the risk for fatal CVD events in patients with T2DM. Of note, agreement between observed and predicted risk improved in the subgroup closest to the derivation cohort of SCORE, i.e. patients without CVD and aged between 40 and 64 years. Similarly, the discriminatory ability for 10-years CVD death improved in this subgroup with a C-index of 0.69, which is similar to SCORE in non-diabetics [5]; however, resulted in the exclusion of more than half of the study population. Since T2DM is particularly a disease of the elderly, age restrictions as given by SCORE would limit its utility in clinical practice.

Previous studies reported that CVD risk scores developed in the general population underestimate risk in individuals with T2DM [34]. Conversely, a brief report by Coleman et al. investigating SCORE in 3898 individuals with newly diagnosed T2DM from the UKPDS cohort reported that SCORE risk equation overestimates the 10-year risk for fatal CVD in individuals with T2DM by 18%, but provides good discriminatory accuracy with a C-index of 0.77 for fatal CVD events [6]. However, the direct comparison of these results with our data may be limited, as the UKPDS included only individuals with newly diagnosed T2DM and treatment has been significantly changed within the study period.

Although much of CV risk can be attributed to traditional risk factors incorporated in classical risk prediction models [35], they do not explain the full spectrum of CVD risk in diabetes [36, 37]. Potential limitations of risk scores calculated at a single point in time may be their inability to account for variability in measured risk factors (e.g. blood pressure), biological variation, exposure duration or untreated risk factor severity. It is not surprising, then, that major CV risk factors are also highly prevalent among individuals who will never experience a CVD event [37]. This paradoxon points to the need for risk assessment tools that allow a more integrated approach at the individual patient level. Intimately tied to this effort will be the requirement to individualize risk beyond the presence of established risk factors.

The current report demonstrates that single NT-proBNP measurement provides a more accurate risk estimate than the newly introduced ESC/EASD risk model. In contrast to both the ESC/EASD model and SCORE, NT-proBNP was even more specific for increased CV risk than all-cause risk in T2DM, emphasizing its clinical relevance as an outcome specific marker.

Yet, numerous studies have indicated that NT-proBNP assessment is effective in refining risk prediction for CVD and adds predictive power to conventional risk models in individuals with T2DM [7–17, 20], only a few studies investigated single NT-proBNP in direct comparison to models composing multiple risk variables [16, 17]. In daily clinical practice, the assessment of a single marker that allows to identify those at highest risk seems attractive. In this study, as in others [18–22], NT-proBNP levels above 125 pg/ml were strongly associated with adverse outcomes in T2DM patients. Given the high negative predictive value of NT-proBNP at a cutoff level of 125 mg/dl [21, 22], initial assessment of NT-proBNP could serve as a first-line screening tool that allows to safely and effectively rule out increased CV risk, while higher values would require further evaluation. In a second approach, additional cardiac investigations may be applied to further refine individual risk. A recent published study conducted in asymptomatic individuals with T2DM reported an additive predictive value of NT-proBNP combined with coronary artery calcium scoring [24]. Similarly, several other studies reported incremental prognostic information of NT-proBNP and troponin T when used in combination [13–15, 23].

The association of NT-proBNP with (CV) hospital admissions observed in this report indicates that NT-proBNP-guided risk stratification may also have the potential for overall cost reductions as already exemplified by previous natriuretic-guided trials in heart failure [38]. The use of NT-proBNP would omit the need for calculation of scores as well as the problem of nonclassification, misclassification or overfitting observed in global risk estimation models.

Yet, two trials provided initial evidence on the effectiveness of natriuretic peptides in guiding preventive efforts in patients at high risk for developing CVD events [19, 39]. In the prospective randomized controlled PONTIAC trial (NT-proBNP selected prevention of cardiac events in a population of diabetic patients without a history of cardiac disease) measurement of NT-proBNP (cut-off 125 pg/ml) was used to identify T2DM patients at high risk for developing CVD [19]. These patients were then stratified to either standard of care treatment or titration for renin-angiotensin inhibitors and beta-blockers. A significant reduction of CVD events was reported in the treatment arm providing initial evidence for a NT-proBNP measurement-based selection of high-risk individuals with T2DM. Similarly, the STOP-HF study (St. Vincent’s Screening to Prevent Heart Failure) demonstrated the effectiveness of natriuretic peptides in guiding preventive efforts in patients with various CV risk factors [39]. A recent observation from the CANVAS (Canagliflozin Cardiovascular Assessment Study) study has shown that canagliflozin treatment in T2DM patients with NT-proBNP levels above 125 pg/ml achieved greater absolute risk reductions in event rates compared to those with lower concentrations [18].

In general, screening for high-risk individuals may only be appropriate when effective treatments are available. The high prevalence of modifiable risk factors among individuals with diabetes but also the recent emerge of new therapies with favorable effects on CVD outcome, such as sodium-glucose cotransporter-2 inhibitors, underscore the importance of identifying those who would most probably benefit from initiation or optimizing treatment.

## Limitations

We are aware of the following limitations of our study. First, retinopathy was not generally documented in this registry and could therefore not be implemented in the risk score which could have led to misclassification. In the current report only 16% of the patients were categorized as moderate or high risk. Hypothetically, the eventual identification of individuals with retinopathy would have led to even more patients being stratified into the very high-risk group, resulting in an even greater weighting of the very high-risk category. A study by Klein et al. demonstrated that retinopathy occurs more frequently in patients with long-term diabetes, CVD and proteinuria [40]. Given the very high risk criteria of the ESC/EASD risk model it seems conceivable that patients with retinopathy may also have been captured in the very high-risk category. Second, as this registry included mainly outpatients followed in hospital, T2DM individuals at lower risk who are more often treated by general practitioners might be underrepresented. In this context, the ESC/EASD model might have performed different with an altered cohort including these patients. Third, survival status was not available in 460 patients after 10 years follow-up. Last, an inverse association between circulating levels of natriuretic peptides and BMI has been reported earlier [41], which could be a potential limitation in a population of T2DM patients. However, despite these observations, natriuretic peptides have been shown to retain prognostic performance in obese patients [42].

## Conclusion

Overall, the current report shows that the recently introduced ESC/EASD risk stratification model provides only limited prognostic information in direct comparison to SCORE and most notably to single NT-proBNP assessment. NT-proBNP measurement is a simple and independent screening tool to identify individuals at increased risk for specifically adverse CVD outcome applicable in a broad spectrum of T2DM patients. Future studies need to investigate the cost-effectiveness and feasibility of NT-proBNP-based screening.

## Supplementary Information

**Additional file 1: Table S1**. ESC/EASD risk stratification in patients with diabetes. **Table S2**. Definitions and specific cut-offs used for the ESC/EASD risk stratification model. **Table S3**. Baseline characteristics presented for the overall cohort and according to the ESC/EASD risk groups. **Table S4**. Competing risk survival analysis for the ESC/EASD model, SCORE and NT-proBNP for the outcomes all-cause death and CVD-death. **Table S5**. Sensitivity, specificity, positive predictive value (PPV) and negative predictive value (NPV) of the risk assessments. **Table S6**. Reclassification table comparing NT-proBNP tertiles with SCORE and the ESC/EASD risk strata. **Table S7**. Predictive information on the predictive performance of i) NT-proBNP when added to a base model including traditional risk factors and ii) the base model when added to NT-proBNP for the outcomes 10-year CVD death and 10-year all-cause death. **Table S8**. Association of the ESC/EASD risk stratification model, SCORE risk and NT-proBNP with outcome in patients without cardiovascular disease (CVD) (n = 1379) and without CVD and aged 40–64 years (n = 707). **Table S9**. Association of NT-proBNP, the ESC/EASD and SCORE risk model with outcome in unselected patients with T2DM (n = 1690). **Figure S1**. Receiver operating characteristic curves of the ESC/EASD CV risk stratification model, SCORE risk estimation and NT-proBNP for the outcomes (A) CV death and (B) all-cause death displayed for the overall cohort (left), T2DM patients without CVD (middle) and without CVD and age 40-64y (right).

## Data Availability

The authors declare that all data supporting the findings of this study are available within the article and its supplementary information files.

## Abbreviations

BMI: Body mass index
CI: Confidence interval
CV: Cardiovascular risk
CVD: Cardiovascular disease
EASD: European Association for the Society of Diabetes
eGFR: Estimated glomerular filtration rate
ESC: European Society of Cardiology
HR: Hazard ratio
IQR: Interquartile range
NRI: Net reclassification improvement
NT-proBNP: N-terminal pro-B-type natriuretic peptide
ROC: Receiver operating characteristic
SCORE: Systemic COronary Risk Evaluation
SE: Standard error
T2DM: Type 2 diabetes mellitus
