# Supplementary material for: Performance of the recommended ESC/EASD cardiovascular risk stratification model in comparison to SCORE and NT-proBNP as a single biomarker for risk prediction in type 2 diabetes mellitus
Source: Cardiovasc Diabetol. 2021 Feb 2;20:34. doi: 10.1186/s12933-021-01221-w (PMC7856811; doi:10.1186/s12933-021-01221-w)
Supplement: Supplementary file 1 — Additional file 1: Table S1. ESC/EASD risk stratification in patients with diabetes. Table S2. Definitions and specific cut-offs used for the ESC/EASD risk stratification model. Table S3. Baseline characteristics presented for the overall cohort and according to the ESC/EASD risk groups. Table S4. Competing risk survival analysis for the ESC/EASD model, SCORE and NT-proBNP for the outcomes all-cause death and CVD-death. Table S5. Sensitivity, specificity, positive predictive value (PPV) and negative predictive value (NPV) of the risk assessments. Table S6. Reclassification table comparing NT-proBNP tertiles with SCORE and the ESC/EASD risk strata. Table S7. Predictive information on the predictive performance of i) NT-proBNP when added to a base model including traditional risk factors and ii) the base model when added to NT-proBNP for the outcomes 10-year CVD death and 10-year all-cause death. Table S8. Association of the ESC/EASD risk stratification model, SCORE risk and NT-proBNP with outcome in patients without cardiovascular disease (CVD) (n = 1379) and without CVD and aged 40–64 years (n = 707). Table S9. Association of NT-proBNP, the ESC/EASD and SCORE risk model with outcome in unselected patients with T2DM (n = 1690). Figure S1. Receiver operating characteristic curves of the ESC/EASD CV risk stratification model, SCORE risk estimation and NT-proBNP for the outcomes (A) CV death and (B) all-cause death displayed for the overall cohort (left), T2DM patients without CVD (middle) and without CVD and age 40-64y (right). [file 12933_2021_1221_MOESM1_ESM.docx]

**SUPPLEMENTARY MATERIAL**

**Supplementary Tables**

**Table S1.** ESC/EASD risk stratification in patients with diabetes.

| Very high risk | Patients with DM **and** established CVD  **or** other target organ damage^*^  **or** three or more risk factors^§^  **or** early onset of T1DM of long duration (>20 years) |
| --- | --- |
| High risk | Patients with DM duration $\geq$10 years without target organ damage  plus any other additional risk factor |
| Moderate risk | Young patients (T1DM aged <35 years or T2DM aged <50 years)  with DM duration <10 years, without other risk factor |
| DM, diabetes mellitus; CVD, cardiovascular disease; T1DM, type 1 diabetes mellitus; T2DM, type 2 diabetes mellitus  * Proteinuria, renal impairment defined as eGFR <30mL/min/1.73m^2^, left ventricular hypertrophy, or retinopathy  § Age, hypertension, dyslipidemia, smoking, obesity | |

**Table S2**. Definitions and specific cut-offs used for the ESC/EASD risk stratification model.

| ESC/EASD risk stratification criteria | |
| --- | --- |
| CVD | |
| history of | CAD, PAD, CeVD, HF, left bundle-branch block, atrial fibrillation and flutter, hypertensive heart disease, valve disorders |
| Organ damage | |
| eGFR | <30mL/min/1.73m^2^ |
| proteinuria | albumin/creatinine ratio >30mg/mmol |
| retinopathy and LVH | not systematically assessed |
| Risk factors | |
| age | >50years |
| obesity | BMI≥30kg/m^2^ |
| hypertension | systolic BP≥140 mmHg and/or diastolic BP≥90 mmHg  and/or current antihypertensive treatment |
| dyslipidaemia | LDL-c ≥116 mg/dL  and/or use of lipid-lowering medications |
| smoking | current smoker |

CAD, coronary artery disease; PAD, peripheral artery disease; CeVD, cerebrovascular disease; HF: heart failure, eGFR, estimated glomerular filtration rate; LVH, left ventricular hypertrophy; BMI, body mass index; BP, blood pressure; LDL, low density lipoprotein

| Table S3. Baseline characteristics presented for the overall cohort and according to the ESC/EASD risk groups. | | | | | | |
| --- | --- | --- | --- | --- | --- | --- |
|  |  | **ESC/EASD CV risk stratification model** | | | | |
|  | **Overall**  **(n=1690)** | **No classification**  **(n=288)** | **Moderate**  **(n=25)** | **High**  **(n=252)** | **Very high**  **(=1125)** | **p-value*** |
| Demographics |  |  |  |  |  |  |
| Age, years (IQR) | 63 [54-69] | 56 [46-67] | 36 [27-41] | 63 [50-72] | 64 [57-70]^***†^ | p<0.001 |
| Female, n (%) | 784 (46) | 134 (47) | 9 (36) | 117 (46) | 524 (47) | p=0.607 |
| Systolic blood pressure, mmHG (IQR) | 142 [130-160] | 138 [125-152] | 123 [111-134] | 140 [125-158] | 145 [131-160]^*** ††^ | p<0.001 |
| Diastolic blood pressure, mmHG (IQR) | 81 [75-90] | 83 [76-92] | 79 [69-86] | 80 [72-88] | 81 [75 - 91]^*††^ | p=0.001 |
| Heart rate, bmp (IQR) | 76 [68-85] | 76 [68-83] | 67 [61-75] | 75 [68-83] | 77 [68-87]^**††^ | p=0.001 |
| Diabetes duration, years (IQR) | 10 [5-19] | 5 [2-8] | 4 [2-8] | 20 [15-29] | 11 [5-19]^***†††^ | p<0.001 |
| Hypertension, n (%) | 1135 (67) | 99 (34) | 0 | 106 (42) | 930 (83)^***†††^ | p<0.001 |
| Dyslipidaemia, n (%) | 1152 (68) | 121 (42) | 0 | 97 (38) | 934 (83)^***†††^ | p<0.001 |
| Smoking, n (%) | 339 (20) | 50 (17) | 0 | 21 (8) | 268 (24)^**†††^ | p<0.001 |
| BMI, kg/m² (IQR) | 28.7 [25.4-32.7] | 26.9 [24.5-29.4] | 23.1 [22.1-26.8] | 26.3 [23.9-28.9] | 30.4 [26.6-34.0]^***†††^ | p<0.001 |
| SCORE, % (IQR) | 8 [4 -12] | 4 [0 - 8] | 0 [0 - 0] | 6 [2 -10] | 8 [4 -12]^***†††^ | p<0.001 |
| Cardiovascular disease |  |  |  |  |  |  |
| PCI, n (%) | 66 (4) | 0 | 0 | 0 | 66 (6)^***†††^ | p<0.001 |
| PAD, n (%) | 173 (10) | 0 | 0 | 0 | 173 (15)^***†††^ | p<0.001 |
| CeVD, n (%) | 99 (6) | 0 | 0 | 0 | 99 (9)^***†††^ | p<0.001 |
| CABG, n (%) | 62 (4) | 0 | 0 | 0 | 62 (6)^***†††^ | p<0.001 |
| Medication |  |  |  |  |  |  |
| Statins, n (%) | 764 (45) | 63 (22) | 0 | 62 (25) | 639 (57)^***†††^ | p<0.001 |
| ASA, n (%) | 634 (38) | 54 (19) | 0 | 84 (33) | 496 (44)^***††^ | p<0.001 |
| Insulin, n (%) | 888 (53) | 107 (37) | 16 (64) | 181 (72) | 584 (52)^†††^ | p<0.001 |
| Metformin, n (%) | 812 (48) | 134 (47) | 6 (24) | 80 (32) | 592 (53)^**†††^ | p<0.001 |
| Sulfonylurea, n (%) | 431 (26) | 81 (28) | 0 (0) | 43 (17) | 307 (27)^***††^ | p<0.001 |
| DPP-4 inhibitor, n (%) | 16 (1) | 1 (0) | 0 (0) | 1 (0) | 14 (0) | p=0.504 |
| GLP-1 agonist, n (%) | 1 (0) | 0 (0) | 0 (0) | 1 (0) | 0 (0) | p=1.000 |
| Glitazone, n (%) | 166 (10) | 19 (7) | 1 (4) | 14 (5) | 132 (12)^††^ | p=0.006 |
| Laboratory parameters |  |  |  |  |  |  |
| NT-proBNP, pg/ml (IQR) | 122 [59-266] | 75 [59-191] | 59 [59-59] | 119 [59-257] | 137 [59-297]^***^ | p<0.001 |
| Albumin/creatinine ratio, mg/mmol (IQR) | 0.87 [0.35-2.94] | 0.72 [0.31-2.09] | 0.55 [0.19-0.94] | 0.57 [0.23-1.92] | 0.90 [0.43-3.99]^*†††^ | p<0.001 |
| Creatinine, mg /dl (IQR) | 0.98 [0.86-1.15] | 0.93 [0.81-1.07] | 0.98 [0.83-1.03] | 0.96 [0.85-1.09] | 1.00 [0.87-1.18]^†^ | p=0.015 |
| eGFR, ml/min (IQR) | 72.7 [60.3-85.3] | 79.0 [66.8-91.2] | 90.1 [83.7-98.5] | 74.2 (62.2-85.9) | 70.3 [57.1-82.2]^***†^ | p<0.001 |
| Triglyceride, mg/dl (IQR) | 129 [91-189] | 123 [89-187] | 98 [69-132] | 89 [68-134] | 141 [100-202]^***†††^ | p<0.001 |
| LDL cholesterol, mg/dl (IQR) | 102 [82-123] | 104 [84-119] | 100 [87-109] | 101 (82-112) | 102 [81-127]^†^ | p=0.051 |
| Cholesterol, mg/dl (IQR) | 187 [162-215] | 186 [166-212] | 182 [151-195] | 188 [165-213] | 188 [161-218] | p=0.261 |
| HbA1c, % (IQR) | 7.2 [6.5-8.1] | 7.0 [6.4-8.1] | 6.7 [6.1-7.4] | 7.3 [6.7-8.0] | 7.2 [6.5-8.1]^§^ | p=0.018 |

Bpm, beats per minute; BMI, body mass index; PCI, percutaneous coronary intervention; PAD, peripheral artery disease; CeVD, cerebrovascular disease; CABG, coronary artery bypass graft; ASA, acetylsalicylic acid; DPP-4, dipeptidyl peptidase-4; GLP-1, glucagon-like peptide 1; NT-proBNP, N-terminal pro-B-type natriuretic peptide; eGFR, estimated glomerular filtration rate; LDL – low-density lipoprotein

*Comparison between the ESC/EASD risk categories moderate, high and very high applying the Kruskal-Wallis and Mann-Whitney U test for continuous variables and Fisher’s exact test for counts. Statistical significance: * or †, respectively, for comparisons of the very high vs. moderate or high risk: ^*^p<0.05, ^**^p<0.01, ^***^p<0.001; ^†^p<0.05, ^††^p<0.01, ^†††^p<0.001

**Table S4.** Competing risk survival analysis for the ESC/EASD model, SCORE and NT-proBNP for the outcomes all-cause death and CVD death.

|  | **All-cause death** |  |  |
| --- | --- | --- | --- |
|  | HR **[**95% CI] | P | C-index [95% CI] |
| **ESC/EASD risk model** |  |  | 0.54 [0.52 to 0.56] |
| **moderate** | reference |  |  |
| **high** | 8.80 [1.21 to 64.00] | 0.032 |  |
| **(high)** x (**CVD death)** | 0.68 [0.40 to 1.17] | 0.168 |  |
| **very high** | 8.62 [1.21 to 61.27] | 0.031 |  |
| **(very high)** x (**CVD death)** | excluded^+^ |  |  |
| **CVD death, 1 = yes, 0 = no** | 0.89 [0.72 to 1.11] | 0.300 |  |
| **SCORE, %^*^** |  |  | 0.63 [0.60 to 0.67] |
| **<5%** | reference |  |  |
| **5-10%** | 2.77 [1.97 to 3.90] | <0.001 |  |
| **(5-10%)** x (**CVD death)** | 1.11 [0.66 to 1.88] | 0.694 |  |
| **>10%** | 2.90 [2.06 to 4.08] | <0.001 |  |
| **(>10%)** x (**CVD death)** | 1.30 [0.77 to 2.18] | 0.325 |  |
| **CVD death, 1 = yes, 0 = no** | 0.70 [0.45 to 1.08] | 0.106 |  |
| **SCORE, %** | 1.05 [1.04 to 1.07] | <0.001 | 0.64 [0.62 to 0.65] |
| **(SCORE)** x (**CVD death)** | 1.00 [0.98 to 1.02] | 0.964 |  |
| **CVD death, 1 = yes, 0 = no** | 0.81 [0.67 to 0.99] | 0.041 |  |
| **NT-proBNP, tertiles** |  |  | 0.70 [0.68 to 0.72] |
| **tertile 1** | reference |  |  |
| **tertile 2** | 1.30 [0.92 to 1.83] | 0.144 |  |
| **(tertile 2)** x (**CVD death)** | 3.27 [1.55 to 6.89] | 0.002 |  |
| **tertile 3** | 2.61 [1.90 to 3.57] | <0.001 |  |
| **(tertile 3)** x (**CVD death)** | 6.52 [3.28 to 12.99] | <0.001 |  |
| **CVD, 1 = yes, 0 = no** | 0.19 [0.10 to 0.36] | <0.001 |  |
| **NT-proBNP, pg/ml^§^** | 2.14 [1.77 to 2.58] | <0.001 | 0.72 [0.70 to 0.74] |
| (**NT-proBNP, pg/ml**) x (**CVD death)** | 2.58 [1.96 to 3.38] | <0.001 |  |
| **CVD death, 1 = yes, 0 = no** | 0.48 [0.38 to 0.62] | <0.001 |  |
| **NT-proBNP, > 125 pg/ml** | 1.92 [1.49 to 2.48] | <0.001 | 0.66 [0.64 to 0.68] |
| **(NT-proBNP, > 125 pg/ml)** x (**CVD death)** | 3.69 [2.32 to 5.86] | <0.001 |  |
| **CVD death, 1 = yes, 0 = no** | 0.31 [0.21 to 0.47] | <0.001 |  |

^*^refers to SCORE treated as categorical variable (cut-off: <5%, 5-10%, >10%)

^§^refers to ln-transformed NT-proBNP per 1-IQR increase

^+^excluded because of a high inter-correlation with CVD death (>0.8)

**Table S5.** Sensitivity, specificity, positive predictive value (PPV) and negative predictive value (NPV) of the risk assessments.

|  | 10-y CV death | | | | 10-year All-cause death | | | |
| --- | --- | --- | --- | --- | --- | --- | --- | --- |
|  | Sensitivity, % | Specifity, % | PPV, % | NPV, % | Sensitivity, % | Specifity, % | PPV, % | NPV, % |
| **Total population** |  |  |  |  |  |  |  |  |
| **ESC/EASD risk model** |  |  |  |  |  |  |  |  |
| Moderate vs. high/very high | 100 | 2 | 13 | 100 | 100 | 2 | 29 | 96 |
| Very high vs. high/moderate | 86 | 21 | 14 | 91 | 83 | 21 | 30 | 76 |
| **SCORE, %** |  |  |  |  |  |  |  |  |
| cut-off 5% | 83 | 42 | 16 | 95 | 81 | 46 | 35 | 87 |
| cut-off 10% | 44 | 72 | 17 | 91 | 42 | 74 | 37 | 78 |
| **NT-proBNP, pg/ml** |  |  |  |  |  |  |  |  |
| cut-off > 125 pg/ml | 85 | 57 | 21 | 97 | 72 | 60 | 39 | 85 |

**Table S6.** Reclassification table comparing NT-proBNP tertiles with SCORE and the ESC/EASD risk strata. Data are given as % row (n).

| **CV mortality** | |  | | **NT-proBNP tertiles** | | | |
| --- | --- | --- | --- | --- | --- | --- | --- |
|  |  | **Risk score** | | **tertile 1** | **tertile 2** | **tertile 3** | **Total** |
|  | No event  (n=1,489) | SCORE | <5% | 53 (328) | 30 (188) | 17 (103) | 100 (619) |
|  |  |  | 5-10% | 29 (128) | 39 (173) | 33 (147) | 100 (448) |
|  |  |  | >10% | 23 (99) | 37 (157) | 39 (166) | 100 (422) |
|  | Event  (n=201) | SCORE | <5% | 11 (4) | 17 (6) | 71 (25) | 100 (35) |
|  |  |  | 5-10% | 5 (4) | 25 (19) | 70 (54) | 100 (77) |
|  |  |  | >10% | 3 (3) | 21 (19) | 75 (67) | 100 (89) |
|  | No event | ESC/EASD model | Moderate | 88 (22) | 8 (2) | 4 (1) | 100 (25) |
|  | (n=1,219) |  | High | 36 (81) | 36 (81) | 29 (65) | 100 (227) |
|  |  |  | Very high | 34 (329) | 35 (343) | 31(295) | 100 (967) |
|  | Event | ESC/EASD model | Moderate | 0 (0) | 0 (0) | 0 (0) | 0 (0) |
|  | (n=183) |  | High | 0 (0) | 32 (8) | 68 (17) | 100 (25) |
|  |  |  | Very high | 4 (7) | 22 (35) | 73 (116) | 100 (158) |
| **All-cause mortality** | | |  |  |  |  |  |
|  | No event  (n=1,242) | SCORE | <5% | 54 (309) | 31 (174) | 15 (86) | 100 (569) |
|  |  |  | 5-10% | 32 (112) | 41 (143) | 27 (94) | 100 (349) |
|  |  |  | >10% | 23 (76) | 40 (130) | 36 (118) | 100 (324) |
|  | Event  (n=448) | SCORE | <5% | 27 (23) | 24 (20) | 49 (42) | 100 (85) |
|  |  |  | 5-10% | 11 (20) | 28 (49) | 61 (107) | 100 (176) |
|  |  |  | >10% | 14 (26) | 25 (46) | 61 (115) | 100 (187) |
|  | No event | ESC/EASD model | Moderate | 88 (21) | 8 (2) | 4 (1) | 100 (24) |
|  | (n=1,001) |  | High | 39 (73) | 37 (69) | 24 (44) | 100 (186) |
|  |  |  | Very high | 36 (287) | 37 (291) | 27 (213) | 100 (791) |
|  | Event | ESC/EASD model | Moderate | 100 (1) | 0 (0) | 0 (0) | 100 (1) |
|  | (n=401) |  | High | 12 (8) | 30 (20) | 58 (38) | 100 (66) |
|  |  |  | Very high | 15 (49) | 26 (87) | 59 (198) | 100 (334) |
| **CVD hospitalization** | | | | | | |  |
|  | No event | SCORE | <5% | 55 (319) | 31 (177) | 14 (81) | 100 (577) |
|  | (n=1,323) |  | 5-10% | 31 (117) | 40 (149) | 29 (107) | 100 (373) |
|  |  |  | >10% | 23 (85) | 38 (140) | 40 (148) | 100 (373) |
|  | Event | SCORE | <5% | 17 (13) | 22 (17) | 61 (47) | 100 (77) |
|  | (n=367) |  | 5-10% | 10 (15) | 28 (43) | 62 (94) | 100 (152) |
|  |  |  | >10% | 12 (17) | 26 (36) | 62 (85) | 100 (138) |
|  | No event | ESC/EASD model | Moderate | 88 (22) | 8 (2) | 4 (1) | 100 (25) |
|  | (n=1,074) |  | High | 37 (78) | 36 (76) | 26 (55) | 100 (209) |
|  |  |  | Very high | 36 (299) | 36 (306) | 28 (235) | 100 (840) |
|  | Event | ESC/EASD model | Moderate | 0 (0) | 0 (0) | 0 (0) | 0 (0) |
|  | (n=328) |  | High | 7 (3) | 30 (13) | 63 (27) | 100 (43) |
|  |  |  | Very high | 13 (37) | 25 (72) | 62 (176) | 100 (285) |
| **All-cause hospitalization** | | | |  |  |  |  |
|  | No event | SCORE | <5% | 59 (185) | 31 (96) | 10 (30) | 100 (311) |
|  | (n=637) |  | 5-10% | 34 (54) | 39 (61) | 27 (43) | 100 (158) |
|  |  |  | >10% | 21 (35) | 38 (64) | 41 (69) | 100 (168) |
|  | Event | SCORE | <5% | 43 (147) | 29 (98) | 29 (98) | 100 (343) |
|  | (n=1,053) |  | 5-10% | 21 (78) | 36 (131) | 43 (158) | 100 (367) |
|  |  |  | >10% | 20 (67) | 33 (112) | 48 (164) | 100 (343) |
|  | No event | ESC/EASD model | Moderate | 82 (14) | 12 (2) | 6 (1) | 100 (17) |
|  | (n=504) |  | High | 46 (51) | 34 (38) | 21 (23) | 100 (112) |
|  |  |  | Very high | 38 (141) | 36 (135) | 26 (99) | 100 (375) |
|  | Event | ESC/EASD model | Moderate | 100 (8) | 0 (0) | 0 (0) | 100 (8) |
|  | (n=898) |  | High | 21 (30) | 36 (51) | 42 (59) | 100 (140) |
|  |  |  | Very high | 26 (195) | 32 (243) | 42 (312) | 100 (750) |

Percentages may not sum to 100% due to rounding

**Table S7**. Predictive performance of i) NT-proBNP when added to a base model including traditional risk factors and ii) the base model when added to NT-proBNP for the outcomes 10-year CVD death and 10-year all-cause death.

|  | Model | C-statistic [95% CI] | p-value | continuous NRI  [95% CI] | p-value |
| --- | --- | --- | --- | --- | --- |
| *10-year CVD death* | | | | | |
|  | Baseline model* | 0.77 [0.74 - 0.80] | - | reference | - |
|  | Baseline model + NT-proBNP | 0.83 [0.80 - 0.85] | 0.004 | 0.28 [0.04 - 0.53] | 0.026 |
|  | NT-proBNP | 0.80 [0.77 - 0.83] | - | reference | - |
|  | NT-proBNP  + Baseline Model | 0.83 [0.80 - 0.85] | ns | 0.72 [0.59 - 0.88] | <0.001 |
| *10-year All-cause death* | | | | | |
|  | Baseline model* | 0.72 [0.70 - 0.75] | - | reference | - |
|  | Baseline model + NT-proBNP | 0.76 [0.74 - 0.78] | 0.011 | 0.17 [0.00 - 0.34] | 0.045 |
|  | NT-proBNP | 0.73 [0.70 - 0.76] | - | reference | - |
|  | NT-proBNP  + Baseline Model | 0.76 [0.74 - 0.78] | ns | 0.69 [0.57 - 0.80] | <0.001 |

*Age, sex, smoking status, cholesterol, systolic blood pressure; ns indicates not significant (p>0.05)

**Table S8.** Association of the ESC/EASD risk stratification model, SCORE and NT-proBNP with outcome in patients without cardiovascular disease (CVD) (n=1379) and without CVD and aged 40-64 years (n=707).

|  |  | Without CVD | | | | | |  | Without CVD, age 40-64y | | | | | |
| --- | --- | --- | --- | --- | --- | --- | --- | --- | --- | --- | --- | --- | --- | --- |
|  | **Predictors** | **HR** | **95%CI** | **P** |  | **C-index** | **95% CI** |  | **HR** | **95%CI** | **P** |  | **C-index** | **95% CI** |
| **10-years**  **CVD death** | **ESC/EASD risk model** | - | - | - |  | 0.51 | 0.47 to 0.55 |  | - | - | - |  | 0.61 | 0.59 to 0.63 |
|  | **moderate** | reference | | |  | - | - |  | reference | | |  | - | - |
|  | **high** | 23.68 | 0.08 to 7013.72 | 0.276 |  | - | - |  |  | N/A** | - |  | - | - |
|  | **very high** | 4.60 | 0.31 to 67.79 | 0.266 |  | - | - |  | 4.55 | 0.02 to 1133.72 | 0.591 |  | - | - |
|  | **SCORE, %** | 1.07 | 1.04 to 1.09 | <0.001 |  | 0.67 | 0.63 to 0.71 |  | 1.08 | 1.04 to 1.13 | <0.001 |  | 0.69 | 0.61 to 0.77 |
|  | **SCORE, %^*^ (<5%, 5-10%, >10%)** | - | - | - |  | 0.64 | 0.59 to 0.68 |  | - | - | - |  | 0.68 | 0.60 to 0.75 |
|  | **<5%** | reference | | |  | - | - |  | reference | | |  | - | - |
|  | **5-10%** | 4.50 | 2.60 to 7.80 | <0.001 |  | - | - |  | 4.45 | 1.98 to 9.98 | <0.001 |  | - | - |
|  | **<10%** | 5.62 | 3.27 to 9.66 | <0.001 |  | - | - |  | 4.52 | 1.84 to 11.12 | 0.001 |  | - | - |
|  | **NT-proBNP, pg/ml**^§^ | 5.26 | 4.16 to 6.65 | <0.001 |  | 0.80 | 0.76 to 0.85 |  | 2.93 | 2.14 to 4.00 | <0.001 |  | 0.74 | 0.65 to 0.83 |
|  | **NT-proBNP, tertiles** | - | - | - |  | 0.75 | 0.72 to 0.79 |  | - | - | - |  | 0.69 | 0.62 to 0.77 |
|  | **tertile 1** | reference | | |  | - | - |  | reference | | |  | - | - |
|  | **tertile 2** | 2.67 | 1.22 to 5.81 | 0.014 |  | - | - |  | 1.96 | 0.63 to 6.09 | 0.242 |  | - | - |
|  | **tertile 3** | 13.38 | 6.75 to 26.51 | <0.001 |  | - | - |  | 5.88 | 2.40 to 14.39 | <0.001 |  | - | - |
|  | **NT-proBNP > 125 pg/ml** | 7.51 | 4.65 to 12.11 | <0.001 |  | 0.72 | 0.68 to 0.75 |  | 4.50 | 2.25 to 9.01 | <0.001 |  | 0.68 | 0.60 to 0.76 |
| **10-years**  **All-cause death** | **ESC/EASD risk model** | - | - | - |  | 0.51 | 0.48 to 0.54 |  | - | - | - |  | 0.54 | 0.50 to 0.58 |
|  | **moderate** | reference | | |  | - | - |  | reference | | |  | - | - |
|  | **high** | 7.45 | 1.03 to 53.65 | 0.046 |  | - | - |  | 1.01 | 0.13 to 7.66 | 0.994 |  | - | - |
|  | **very high** | 2.65 | 0.99 to 7.08 | 0.052 |  | - | - |  | 1.29 | 0.48 to 3.45 | 0.614 |  | - | - |
|  | **SCORE, %** | 1.06 | 1.05 to 1.08 | <0.001 |  | 0.67 | 0.64 to 0.70 |  | 1.07 | 1.04 to 1.09 | <0.001 |  | 0.66 | 0.61 to 0.71 |
|  | **SCORE, %^*^ (<5%, 5-10%, >10%)** | - | - | - |  | 0.63 | 0.60 to 0.67 |  | - | - | - |  | 0.63 | 0.58 to 0.68 |
|  | **<5%** | reference | | |  | - | - |  | reference | | |  | - | - |
|  | **5-10%** | 3.43 | 2.51 to 4.69 | <0.001 |  | - | - |  | 2.45 | 1.60 to 3.75 | <0.001 |  | - | - |
|  | **<10%** | 3.82 | 2.80 to 5.21 | <0.001 |  | - | - |  | 3.15 | 1.98 to 5.00 | <0.001 |  | - | - |
|  | **NT-proBNP, pg/ml**^§^ | 2.91 | 2.51 to 3.38 | <0.001 |  | 0.71 | 0.68 to 0.74 |  | 2.05 | 1.71 to 2.47 | <0.001 |  | 0.66 | 0.61 to 0.72 |
|  | **NT-proBNP, tertiles** | - | - | - |  | 0.68 | 0.65 to 0.71 |  | - | - | - |  | 0.63 | 0.58 to 0.69 |
|  | **tertile 1** | reference | | |  | - | - |  | reference | | |  | - | - |
|  | **tertile 2** | 1.54 | 1.09 to 2.17 | <0.014 |  | - | - |  | 0.99 | 0.55 to 1.77 | 0.986 |  | - | - |
|  | **tertile 3** | 4.06 | 3.02 to 5.74 | <0.001 |  | - | - |  | 2.79 | 1.84 to 4.23 | <0.001 |  | - | - |
|  | **NT-proBNP > 125 pg/ml** | 2.97 | 2.35 to 3.76 | <0.001 |  | 0.65 | 0.62 to 0.68 |  | 2.84 | 2.00 to 4.10 | <0.001 |  | 0.64 | 0.58 to 0.69 |
| **5-years CV hospitalization** | **ESC/EASD risk model** | - | - | - |  | 0.52 | 0.49 to 0.55 |  | - | - | - |  | 0.54 | 0.50 to 0.58 |
|  | **moderate** | reference | | |  | - | - |  | reference | | |  | - | - |
|  | **high** | 23.63 | 0.30 to 1860.35 | 0.156 |  | - | - |  | 22.46 | 0.00 to 262756.96 | 0.515 |  | - | - |
|  | **very high** | 4.60 | 0.60 to 35.02 | 0.141 |  | - | - |  | 4.55 | 0.10 to 205.77 | 0.436 |  | - | - |
|  | **SCORE, %** | 1.05 | 1.04 to 1.07 | <0.001 |  | 0.67 | 0.61 to 0.68 |  | 1.06 | 1.03 to 1.09 | <0.001 |  | 0.67 | 0.62 to 0.73 |
|  | **SCORE, %^*^ (<5%, 5-10%, >10%)** | - | - | - |  | 0.61 | 0.58 to 0.64 |  | - | - | - |  | 0.62 | 0.56 to 0.67 |
|  | **<5%** | reference | | |  | - | - |  | reference | | |  | - | - |
|  | **5-10%** | 2.99 | 2.11 to 4.24 | <0.001 |  | - | - |  | 2.53 | 1.59 to 4.02 | <0.001 |  | - | - |
|  | **<10%** | 2.79 | 1.95 to 3.99 | <0.001 |  | - | - |  | 2.62 | 1.55 to 4.45 | <0.001 |  | - | - |
|  | **NT-proBNP, pg/ml**^§^ | 3.38 | 2.85 to 4.01 | <0.001 |  | 0.74 | 0.70 to 0.77 |  | 2.42 | 1.97 to 2.96 | <0.001 |  | 0.70 | 0.64 to 0.77 |
|  | **NT-proBNP, tertiles** | - | - | - |  | 0.70 | - |  | - | - | - |  | 0.67 | 0.61 to 0.72 |
|  | **tertile 1** | reference | | |  | - | - |  | reference | | |  | - | - |
|  | **tertile 2** | 2.13 | 1.36 to 3.34 | 0.001 |  | - | - |  | 1.60 | 0.85 to 3.04 | 0.148 |  | - | - |
|  | **tertile 3** | 6.10 | 4.09 to 9.09 | <0.001 |  | - | - |  | 3.91 | 2.37 to 6.45 | <0.001 |  | - | - |
|  | **NT-proBNP > 125 pg/ml** | 3.56 | 2.67 to 4.76 | <0.001 |  | 0.66 | 0.63 to 0.70 |  | 3.30 | 2.18 to 4.98 | <0.001 |  | 0.65 | 0.60 to 0.71 |
| **5-years**  **All-cause hospitalization** | **ESC/EASD risk model** | - | - | - |  | 0.53 | 0.51 to 0.56 |  | - | - | - |  | 0.55 | 0.52 to 0.59 |
|  | **moderate** | reference | | |  | - | - |  | reference | | |  | - | - |
|  | **high** | 2.18 | 1.07 to 4.44 | 0.033 |  | - | - |  | 1.47 | 0.46 to 4.71 | 0.519 |  | - | - |
|  | **very high** | 1.57 | 1.11 to 2.23 | 0.011 |  | - | - |  | 1.47 | 0.83 to 2.60 | 0.185 |  | - | - |
|  | **SCORE, %** | 1.03 | 1.02 to 1.04 | <0.001 |  | 0.60 | 0.57 to 0.63 |  | 1.03 | 1.01 to 1.05 | 0.001 |  | 0.59 | 0.55 to 0.63 |
|  | **SCORE, %^*^ (<5%, 5-10%, >10%)** | - | - | - |  | 0.55 | 0.53 to 0.56 |  | - | - | - |  | 0.54 | 0.52 to 0.57 |
|  | **<5%** | reference | | |  | - | - |  | reference | | |  | - | - |
|  | **5-10%** | 1.59 | 1.35 to 1.88 | <0.001 |  | - | - |  | 1.53 | 1.22 to 1.93 | 0.001 |  | - | - |
|  | **<10%** | 1.51 | 1.27 to 1.79 | <0.001 |  | - | - |  | 1.48 | 1.13 to 1.94 | 0.005 |  | - | - |
|  | **NT-proBNP, pg/ml**^§^ | 1.53 | 1.38 to 1.70 | <0.001 |  | 0.60 | 0.57 to 0.63 |  | 1.32 | 1.17 to 1.50 | 0.001 |  | 0.56 | 0.52 to 0.60 |
|  | **NT-proBNP, tertiles** | - | - | - |  | 0.59 | 0.56 to 0.62 |  | - | - | - |  | 0.55 | 0.51 to 0.59 |
|  | **tertile 1** | reference | | |  | - | - |  | reference | | |  | - | - |
|  | **tertile 2** | 1.21 | 1.01 to 1.45 | 0.035 |  | - | - |  | 0.96 | 0.73 to 1.25 | 0.746 |  | - | - |
|  | **tertile 3** | 1.70 | 1.44 to 2.01 | <0.001 |  | - | - |  | 1.32 | 1.05 to 1.65 | 0.016 |  | - | - |
|  | **NT-proBNP > 125 pg/ml** | 1.43 | 1.24 to 1.64 | <0.001 |  | 0.56 | 0.54 to 0.59 |  | 1.34 | 1.09 to 1.65 | 0.006 |  | 0.55 | 0.52 to 0.59 |

^*^refers to SCORE as categorical variable (cut-off: <5%, 5-10%, >10%), ^§^refers to ln-transformed NT-proBNP per 1-IQR increase ^**^not calculable as no events occurred

**Table S9.** Association of NT-proBNP, the ESC/EASD and SCORE risk model with outcome in unselected patients with T2DM (n=1690).

|  | 5-y Cardiovascular hospitalization | | |  | 5-y All-cause hospitalization | | |
| --- | --- | --- | --- | --- | --- | --- | --- |
|  | HR **[**95% CI] | P | C-index [95% CI] |  | HR **[**95%CI] | P | C-index [95% CI] |
| **ESC/EASD risk model** | - | - | 0.54 [0.52 to 0.56] |  | - | - | 0.55 [0.52 to 0.57] |
| **moderate** | reference | | - |  | reference | | - |
| **high** | 23.63 [0.30 to 1860.35] | 0.156 | - |  | 2.18 [1.07 to 4.44] | 0.033 | - |
| **very high** | 4.57 [0.90 to 23.35] | 0.068 | - |  | 1.72 [1.21 to 2.43] | 0.002 | - |
| **SCORE, %** | 1.05 [1.04 to 1.06] | <0.001 | 0.62 [0.59 to 0.65] |  | 1.03 [1.02 to 1.04] | <0.001 | 0.59 [0.56 to 0.62] |
| **SCORE, %^*^**  **(<5%, 5-10%, >10%)** | - | - | 0.60 [0.57 to 0.62] |  | - | - | 0.62 [0.60 to 0.64] |
| **<5%** | reference | | - |  | reference | | - |
| **5-10%** | 2.71 [2.06 to 3.56] | <0.001 | - |  | 1.53 [1.32 to 1.77] | <0.001 | - |
| **<10%** | 2.59 [1.96 to 3.42] | <0.001 | - |  | 1.46 [1.26 to 1.70] | <0.001 | - |
| **NT-proBNP, pg/ml**^§^ | 3.53 [3.06 to 4.07] | <0.001 | 0.74 [0.71 to 0.77] |  | 1.62 [1.48 to 1.78] | <0.001 | 0.62 [0.59 to 0.64] |
| **NT-proBNP, tertiles** | - | - | 0.71 [0.68 to 0.74] |  | - | - | 0.61 [0.58 to 0.64] |
| **tertile 1** | reference | | - |  | reference | | - |
| **tertile 2** | 2.27 [1.60 to 3.24] | <0.001 | - |  | 1.28 [1.10 to 1.50] | 0.002 | - |
| **tertile 3** | 6.39 [4.64 to 8.81] | <0.001 | - |  | 1.86 [1.60 to 2.16] | <0.001 | - |
| **NT-proBNP, > 125 pg/ml** | 3.46 [2.74 to 4.36] | <0.001 | 0.66 [0.63 to 0.69] |  | 1.50 [1.33 to 1.70] | <0.001 | 0.58 [0.56 to 0.60] |

^*^refers to SCORE as categorical variable (<5%, 5-10%, >10%), ^§^refers to ln-transformed NT-proBNP per 1-IQR increase

**Supplementary Figures**

**Figure S1.** Receiver operating characteristic curves of the ESC/EASD CV risk stratification model, SCORE risk estimation and NT-proBNP for the outcomes (A) CV death and (B) all-cause death displayed for the overall cohort (left), T2DM patients without CVD (middle) and without CVD and age 40-64y (right).
